# Metamorphosis of Benthic Invertebrate Larvae: A Sensitive Indicator for Detection of Changes in Marine Environmental Quality

**DOI:** 10.1100/tsw.2002.123

**Published:** 2002-02-22

**Authors:** Xuelei Zhang

**Affiliations:** Key Lab for Science and Engineering of Marine Ecological Environment, First Institute of Oceanography, State Oceanic Administration, Qingdao 266061, China

**Keywords:** bioassay, metamorphosis, pollution, toxicology

## Abstract

Many marine coastal ecosystems are threatened by increasing waste discharge, and bioassays are needed because chemical and physical tests alone are not sufficient to assess potential effects on aquatic biota. In light of the fact that larval metamorphosis of benthic invertebrates is usually very sensitive to environmental changes, this paper reviews sensitivity comparisons of larval metamorphosis with other common bioassays in different species and those in different developmental stages of the same species, and it discusses the potential use of larval metamorphosis as an indicator of marine environmental quality.

## INTRODUCTION

The health and well being of many marine coastal ecosystems are threatened by increasing industrial, agricultural, and municipal waste discharges. To assess these threats, proper bioassays are needed because chemical and physical tests alone are not sufficient to assess potential effects on aquatic biota[1]. Indicator selection is central for toxicity tests that are used for the determination of the safety levels of pollutants and for setting up environmental standards. Various acute or chronic tests have been developed, including biochemical (enzymatic activity, ATP/AMP ratio, etc.), genetic (micronuclei), pathological (tumors), physiological (photosynthesis, respiration, feeding, etc.), behavioral (escaping, burrowing, etc.) and reproductive (spawning fecundity, fertilization) responses, developmental/growth abnormality, mortality, as well as population and community structure. Among these tests, only a few have been adopted widely as standard methods. Fish tests (mortality, escaping behavior, reproduction, metabolism, and growth) were the earliest adopted standards, but their cost and complexity hindered their application. Tests using the early stages of benthic invertebrates, such as sea urchin fertilization or bivalve embryogenesis, were also adopted and are still being used in North American, European, and some Asian countries[2]. Recently, microbial assays (e.g., bacterial bioluminescence)[3], which can be automated for rapid screening of toxicants, have also been adopted as standard methods. However, it is noted that a more in-depth toxicological analysis requires inclusion of bioassays with invertebrates because of their higher discriminatory power[4,5].

In light of the problems with the existing bioassay methods, there has been a continued search for new assay methods, of which most deal with marine invertebrates[2,6,7]. These studies have indicated that larval metamorphosis of benthic invertebrates is particularly sensitive to environmental changes[8,9,10]. Metamorphosis is the morphological and behavioral transition process from a larval invertebrate to a juvenile. Current studies indicate that pollutants can interfere with larval metamorphosis in several benthic invertebrates. While the tumor-promoting phorbol esters facilitate metamorphosis in the hydrozoans, many other pollutants (zinc, copper, cadmium, chromium, tributyltin, alcohols, phenols, DDT, and hydrocarbons) inhibit metamorphosis in the hydrozoans, oysters, scallops, abalones, the chitons, and sea urchins[11,12,13,14,15,16,17,18,19,20,21,22].

This paper reviews sensitivity comparisons for larval metamorphosis with other common assays in different species and those in different developmental stages of the same species, and it discusses the potential use of larval metamorphosis as an indicator of marine environmental quality.

## METHODOLOGY

Median effective concentrations (EC50) or lethal concentrations (LC50) of various pollutants in invertebrates from broad taxonomic categories, or lowest effective concentrations (LOEC) where EC50/LC50 is not available, were cited to compare sensitivity of assay methods using different indicators from different species or various development stages of the same species. No statistical analyses were conducted due to the use of different experimental protocols.

## RESULTS

The sensitivity of larval metamorphic tests varies, either in the same range of or lower than EC50/LC50 determined with other common tests (Table 1). Specifically, larval metamorphosis tests are as sensitive as common tests for organotin (TBT) and volatile organic chemicals (ethanol, butanol, and pentanol). However, larval metamorphosis assays are slightly more sensitive to heavy metals ($Cu^{2+}$, $Zn^{2+}$, and $Cd^{2+}$). Moreover, metamorphosis is tens to hundreds of times more sensitive to organochlorine (DDT) than are other indicators.

When comparative studies are made in the same species, larval metamorphosis is, more than often, more sensitive than are other elements of the life history (Table 2). Among the available data, metamorphosis of molluscs (bay scallop *Argopecten irradians*, Pacific oyster *Crassostrea gigas*, and red abalone *Haliotis rufescens*) is more sensitive to toxicants than are other life stages. Metamorphosis is less or as sensitive as other early life stages in the sea urchin *Heliocidaris erythrogramma* (to $Cu^{2+}$) as well as in the red abalone *Haliotis rufescens* (to DDT).

## DISCUSSION

Toxicity tests have two prime requirements for indicators: high sensitivity and significant ecological relevance. In addition, they should be cost effective and easy to examine indoors or out in the field. Metamorphosis assays clearly meet these requirements. The data shown above indicate the merit of larval metamorphic tests in detecting lower levels of heavy metals and the

**TABLE 1**
**Toxicity of Some Chemicals (mg/dm$^3$) to Various Aquatic Organisms**

| | Test \ Chemical | Ethanol | 1-Butanol | 1-Pentanol | DDT | TBT | $Cu^{2+}$ | $Zn^{2+}$ | $Cd^{2+}$ | Ref. |
|---|---|---|---|---|---|---|---|---|---|---|
| Metamorphosis | Cnidarian larvae 3-h EC50 | $4.3 \times 10^4$ | $7.5 \times 10^3$ | $2.1 \times 10^3$ | | $1.1 \times 10^{-3}$ | | | | 11 |
| | *Chironomus* sp. larvae 10-day EC50 | | | | | $9.96 \times 10^{-3}$ | | | | 22 |
| | Chiton larvae 96-h LOEC | | | | | | 0.002 | 0.002 | 0.002 | 20 |
| | Oyster larvae 20-day EC50 | | | | | | 0.035 ~0.045 | 0.030 ~0.035 | 0.020 ~0.025 | 21 |
| | Abalone larvae 15-h EC50 | | | | 0.05 | | | | | 17 |
| Other Tests | Microbial biodegradation inhibition 75-min EC50 | $3.3 \times 10^4$ | $1.1 \times 10^4$ | $5.4 \times 10^4$ | | | | | | 23 |
| | Copepod 96-h LC50 | $7.6 \times 10^3$ | $2.1 \times 10^3$ | 436 | | | | | | 24 |
| | Fathead minnow 96-h LC50 | $1.5 \times 10^4$ | $1.7 \times 10^3$ | 602 | | | | | | 25 |
| | *Artemia* larvae 72-h LC50 | | | | 16.4 | | | | | 26 |
| | Sheephead minnow 7-day LC50 | | | | | $5 \times 10^{-3}$ | | | | 27 |
| | *Daphnia* sp. 48-h LC50 | | | | | $2 \times 10^{-3}$ | | | | 27 |
| | Oyster embryo 48-h LC50 | | | | | | 0.13 | 0.34 | 3.8 | 28 |
| | *Penaeus* sp. 48-h LC50 | | | | | | 0.14 | 0.84 (96-h) | 0.46 | 29 |
| | Various marine organisms 48-h LC50 | | | | $4 \times 10^{-3}$ ~1.0 | | $10^{-3}$ ~0.1 | 0.01 ~0.1 | 0.01 ~1.0 | 29 |

disadvantage of their not being sensitive to volatile toxicants. Further, metamorphosis is in most cases more sensitive than other life stages of benthic invertebrates, as metamorphosis is a well-known bottleneck of aquatic invertebrates. Failure in metamorphosis and settlement means failure in recruitment to the population[30,31,32]. The metamorphic assays thus have clear ecological relevance. Additionally, the rapidly increasing body of metamorphic research has revealed dramatic and subtle behavioral and morphological changes during metamorphosis and their stimuli in short exposure periods[33,34]. Hence, it is easy and cost effective to observe any interference effects simply with several incubating vials containing small volumes of test media and a dissecting microscope.

Of the many benthic invertebrates, larvae of polychaetes, barnacles, and bryozoans are the best candidates for larval metamorphosis assays. They have short life histories (i.e., quick maturation and frequent spawning); their microalgae-based culture and reproductive control have been well established in many labs around the world. Hence, their larvae are available everywhere throughout the year and researchers are able to integrate larval study with studies of other developmental stages to cover the entire life history within the same species. Furthermore, the short duration (a couple of hours to several days) before these larvae become competent to metamorphose and the relatively short duration (usually less than 2 days) for metamorphosis to complete make the assays easy to manipulate. In fact, larvae of these organisms have been

**TABLE 2**
**Toxicity of Chemicals ($mg/dm^3$) to Different Life Stages of Marine Invertebrates**

| Organism | Chemical | Toxicity: EC50 or LC50 (Duration of Exposure) | Ref. |
|---|---|---|---|
| Bay scallop (*Argopecten irradians*) | $CrO_4^{2-}$ | Zygote hatching: 2.61 (48 h)<br>Straight-shelled larvae: 3.89 (48 h)<br>Hinge-shelled larvae: 2.40 (48 h)<br>Metamorphosis: 0.83 (6 day) | 16 |
| Oyster (*Crassostrea gigas*) | $Cu^{2+}$ (spiked in sediment interstitial water) | Larval mortality: 300 (96 h)<br>Metamorphosis: 50 (96 h) | 19 |
| Red abalone (*Haliotis rufescens*) | $Zn^{2+}$ | Larval shell development: 40 (48 h)<br>Metamorphosis: 34 (48 h) | 12 |
| | Bleach effluent | Larval shell development: 0.98% (48 h)<br>Metamorphosis: 0.76% (48 h) | 12 |
| | $Cu^{2+}$ | Larval mortality: 3.2 (15 h)<br>Metamorphosis: 0.064 (15 h) | 17 |
| | DDT | Larval mortality: 0.05 (15 h)<br>Metamorphosis: >1.0 (15 h) | 17 |
| Sea urchin (*Heliocidaris erythrogramma*) | $Cu^{2+}$ | Sperm viability: 0.001 (3 min)<br>Fertilization: 0.01 (3 min)<br>Cleavage: 0.02 (30 min)<br>Blastula: 0.01 (10 h)<br>Gastrula: 0.005 (15 h)<br>Metamorphosis: 0.005 (60 h) | 15 |

employed in antifoulant screening for a long time[35,36,37]. Meanwhile, larvae of these species are among the best studied in metamorphic investigations[38,39], and discovering underlying mechanisms should assist in toxicological research. For these reasons, further studies should lead to the adoption of larval metamorphosis assays using polychaetes, barnacles, and bryozoan larvae in the testing of toxicants in marine environments.

## ACKNOWLEDGEMENT

This work was supported with the National Natural Science Fund (No. 49906009) and State Oceanic Administration Fund to Young Scientists (No. 2000304). The author thanks Dr. J.W. Qiu and an anonymous reviewer for helpful comments on the manuscript.
